# High pretreatment peripheral blood T‐cell receptor clonality as a predictor of prolonged response in immune thrombocytopenia

**DOI:** 10.1111/bjh.70310

**Published:** 2026-01-04

**Authors:** Paul Schmidt‐Barbo, Christoph Schultheiss, Annaïse J. Jauch, Donjetë Simnica, Gerda Silling, Mathias Hänel, Jörg Chromik, Thomas Stauch, Karolin Trautmann‐Grill, Roland Repp, Clemens Schulte, Bastian Fleischmann, Manfred Welslau, Martina Stauch, Claudia Quiering, Frank Richter, Tamara Tesanovic, Sabine Jahn, Andreas Holbro, Jakob R. Passweg, Axel Matzdorff, Mathias Rummel, Oliver Meyer, Falk Nimmerjahn, Mascha Binder

**Affiliations:** ^1^ Division of Medical Oncology University Hospital Basel Basel Switzerland; ^2^ Laboratory of Translational Immuno‐Oncology, Department of Biomedicine University and University Hospital Basel Basel Switzerland; ^3^ Collaborative Research Institute Intelligent Oncology (CRIION) Freiburg Germany; ^4^ Division of Haematology University Hospital Basel Basel Switzerland; ^5^ Division of Clinical Pharmacology, Ludwig Maximilian University Hospital Ludwig Maximilian University Munich Munich Germany; ^6^ Department of Haematology, Oncology, Haemostaseology and Stem Cell Transplantation, Faculty of Medicine RWTH Aachen University Aachen Germany; ^7^ Department of Internal Medicine III Klinikum Chemnitz Chemnitz Germany; ^8^ Department of Medicine II, Haematology and Oncology, Goethe University Frankfurt University Hospital Frankfurt Germany; ^9^ Department of Internal Medicine II, Haematology and Oncology University Hospital Jena Jena Germany; ^10^ Department of Internal Medicine I University Hospital Dresden Dresden Germany; ^11^ Medical Department 2 City Hospital Kiel Kiel Germany; ^12^ Gemeinschaftspraxis für Hämatologie und Onkologie Dortmund Germany; ^13^ Onkologisches Zentrum Donauwörth Donauwörth Germany; ^14^ Onkologie Aschaffenburg Aschaffenburg Germany; ^15^ Haematology, Oncology/Haemostaseology Kronach Kronach Germany; ^16^ Novartis Pharma GmbH Nürnberg Germany; ^17^ Department of Medicine II Asklepios Clinic Uckermark Schwedt Germany; ^18^ Department of Haematology, Clinic for Haematology and Medical Oncology Justus Liebig University Hospital Giessen Giessen Germany; ^19^ DRK Blood Service NSTOB Springe Germany; ^20^ Division of Genetics, Department of Biology Friedrich Alexander University Erlangen‐Nürnberg Erlangen Germany

**Keywords:** BCR, biomarker, eltrombopag, immune repertoire, ITP, TCR

## Abstract

Left panel: Scheme of the XPAG‐immune thrombocytopenia trial. The dexamethasone (DEX) arm consisted of DEX 40 mg/day for days 1–4 for one to three cycles every 28 days to a maximum of 12 weeks, cycles 2 + 3 were optional. Patients randomised to eltrombopag (ETB) + DEX received eltrombopag in combination with a short course of high‐dose DEX beginning on day 1 (40 mg/day during days 1–4). The starting dose of ETB was 50 mg/day for 2 weeks; thereafter, the ETB dose was increased by 25 mg for all patients who did not achieve the target platelet count of ≥50 × 10^9^/L. The ETB tapering was performed by decreasing the dose by 25 mg every 2 weeks to a minimum dose of 25 mg every other day for all patients. Right upper panel: Clinical outcomes in patients treated with first‐line ETB + DEX. Patients displayed a qualitatively longer response duration and qualitatively reduced usage of rescue medication. Right lower panel: The immunological T‐cell response was different in treatment responders and non‐responders. Patients with sustained response (responders) displayed a high T‐cell clonality at baseline. Clones are depicted as bubbles (right side).

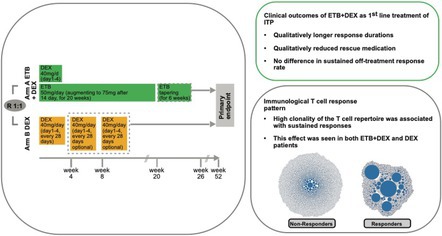

To the Editor,

Primary immune thrombocytopenia (ITP) is an autoimmune disease characterised by reduced platelet counts and increased bleeding risk which can manifest as bruises, petechiae or, in few patients, as severe and fatal haemorrhage^1^, ^2^ The pathogenesis of ITP is considered multifactorial involving autoantibody‐mediated platelet depletion and impaired thrombopoietin (TPO)‐regulated platelet production by megakaryocytes.^2^ In addition, the dysregulation of the T‐cell compartment is increasingly recognised.^3^ Loss of T‐cell tolerance can prime B cells to produce anti‐platelet antibodies or trigger T‐cell‐mediated apoptosis of megakaryocytes, thus blocking platelet production.^2^ These features are also reflected in skewed T helper compartments and the identification of ITP‐predisposing genetic polymorphisms in T‐cell costimulatory factors, cytokines and Fc gamma receptors.^1^ Although immunosuppression by corticosteroids remains the main first‐line treatment, this approach does not offer long‐term remission and comes with significant side effects. To address these limitations, recombinant TPO‐mimetics were developed to boost platelet production. However, development was halted when patients developed anti‐TPO antibodies that cross‐reacted with endogenous TPO, leading to severe thrombocytopenia.^4^ As non‐autoreactive alternative, thrombopoietin receptor agonists (TPO‐RAs) are now widely used for ITP management. The practice of tapering TPO‐RAs to maintain remission only became standard in subsequent years, allowing one‐third of treated patients to remain in remission after tapering.^5^, ^6^

Eltrombopag (ETB)^7^, ^8^ is a widely used TPO‐RA that stimulates megakaryopoiesis and counteracts the reduced endogenous TPO activity observed in ITP. While it is well established in the management of chronic and refractory ITP, its role in the initial treatment setting is less well defined. We performed a phase II randomised trial to evaluate ETB in combination with a single course of high‐dose dexamethasone (ETB + DEX) compared to dexamethasone alone (DEX) in adults with newly diagnosed ITP (XPAG‐ITP trial),^9^ published back to back with this letter. Overall and complete response rates were similar across both arms, but median OR duration was longer in the ETB + DEX arm and the application of rescue medication was reduced (Jauch et al., BJH 2025).

To better understand the immunological effects of treatment, we profiled the peripheral T‐cell receptor (TCR) and B‐cell receptor (BCR) repertoires in both treatment arms. Immune repertoire data from healthy donors (HDs) served as controls.^10^, ^11^, ^12^, ^13^ A detailed description of the methods used can be found in the supplemental material. Analysis of repertoire metrics revealed trends of higher baseline clonality and richness in the T‐cell compartment of both treatment groups (Figure 1A,B). The BCR repertoire also showed trends towards higher richness (Figure 1C,D), but no clear imprint of antigen selection (Figure 1E). The levels of somatic hypermutation indicate rather naïve BCR repertoires as compared to HD (Figure 1E). Importantly, when patients with sustained response off‐treatment at week 52 were compared to the remaining patients, we noted important differences in their clonal T‐cell spaces. Patients with sustained responses showed more clonal T‐cell repertoires prior to treatment initiation with a high percentage of large and hyperexpanded clones. In these responders, the percentage of large clones decreased over the treatment course (Figure 1F). The diversification of the T‐cell repertoire observed in both ETB‐DEX and DEX responders suggests an immunological response pattern that is not specific for the treatment regimen, but rather characteristic of ITP pathobiology. To further explore this, we interrogated our TCR dataset for shared CDR3 clonotypes that might reflect common autoreactive T cells driving platelet destruction. However, we did not detect a high degree of inter‐patient clonal overlap (Figure 2A). Next, we investigated the T‐cell receptor beta‐chain variable (TRBV) and TRBV‐J gene usage architectures in ITP patients. As shown in Figure 2B, the TCR repertoires of ITP patients were highly skewed independent of treatment regimen or cycle. Within each treatment group, the degree of skewing was similar for V and V‐J gene usage indicating TRBV usage rather than selection of distinct rearrangements as dominant driver of ITP T‐cell architecture (Figure 2B). Notably, while the overall skewing patterns for V or V‐J gene usage were similar between treatment groups, the ETB + DEX group appears to have lower degrees of statistical TCR skewing (Figure 2B). When analysing the underlying TRBV frequency pattern, we noticed an enrichment of TRBV12‐3 and a lack of TRBV6‐2 and TRBV12‐4 rearrangements independent of treatment (Figure 2C). Patients in the ETB + DEX arm appeared to have a slight increase of TRBV7‐9, TRBV20‐1 and TRBV12‐3 rearrangements at W27 and W53 (Figure 2C).

**FIGURE 1:**
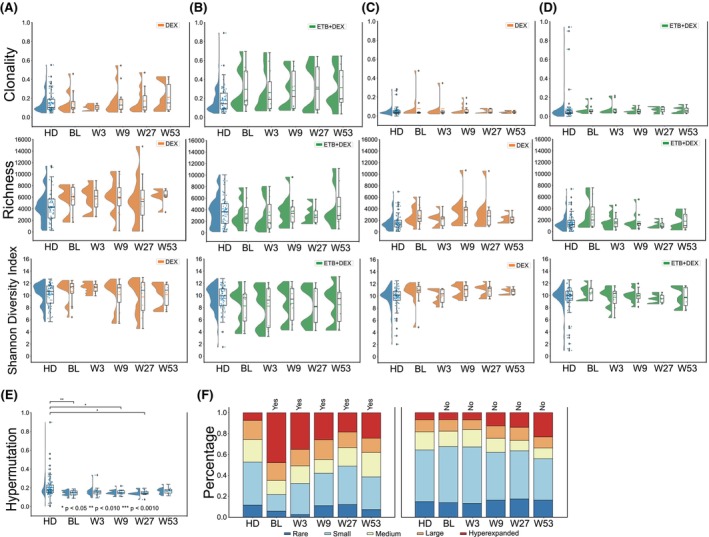
Immune repertoire metrics in the XPAG‐ITP cohort compared to healthy individuals. T‐cell repertoire clonality, richness and diversity in healthy individuals HD (*n* = 106) and XPAG‐ITP patient population (BL, W3, W9, W27, W53) are shown over the course of treatment in the DEX (A, orange) and the DEX + ETB arm (B, green). Cohort size: BL (*n* = 12 for DEX, *n* = 8 for ETB + DEX), W3 (*n* = 10 for DEX, *n* = 9 for ETB + DEX), W9 (*n* = 11 for DEX, *n* = 10 for ETB + DEX), W27 (*n* = 9 for DEX, *n* = 7 for ETB + DEX) and W53 (*n* = 6 for DEX, and *n* = 6 for ETB + DEX). B‐cell repertoire clonality, richness and diversity in age‐matched healthy individuals and XPAG‐ITP patient population are shown over the course of treatment in the DEX (C) and the DEX + ETB arm (D). Cohort size: BL (*n* = 12 for DEX, *n* = 8 for ETB + DEX), W3 (*n* = 8 for DEX, *n* = 11 for ETB + DEX), W9 (*n* = 11 for DEX, *n* = 10 for ETB + DEX), W27 (*n* = 9 for DEX, *n* = 6 for ETB + DEX) and W53 (*n* = 7 for DEX, and *n* = 6 for ETB + DEX). (E) Fraction of antigen‐experienced B cells are shown as percentage of cells with somatic *IGH* hypermutation within the repertoire for both treatment arms. (F) Clonal T spaces in patients with or without sustained response off treatment at week 52. T‐cell spaces in patients with (Yes; Cohort size: *n* = 3 for BL, *n* = 2 for W3, *n* = 4 for W9, *n* = 3 for W27, *n* = 2 for W53, *n* = 37 for HD) or without (No; Cohort size: *n* = 17 for BL, *n* = 17 for W3, *n* = 17 for W9, *n* = 13 for W27, *n* = 10 for W53, *n* = 84 for HD) sustained responses in comparison to a sex‐ and age‐matched control cohort. BL, baseline; DEX, dexamethasone; ETB, eltrombopag; HD, healthy donor; IGH, immunoglobulin heavy chain; W3, week 3; W9, week 9; W27, week 27; W53, week 53.

**FIGURE 2:**
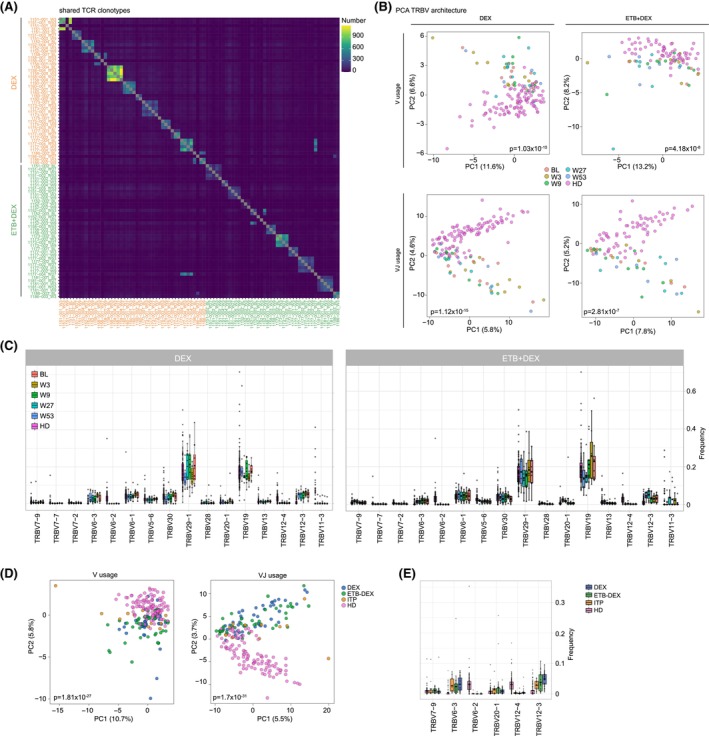
T‐cell receptor (TCR) architecture in ITP patients. (A) Inter‐ and intra‐patient clonal overlap of unique TCR CDR3 clonotypes of the XPAG‐ITP (DEX, ETB + DEX) cohort and an independent ITP cohort^11^ is displayed as a heat map. (B) PCA of TRBV and TRBV‐J gene usage in the XPAG‐ITP cohort depending on the treatment arm (DEX vs. ETB + DEX) as compared to healthy individuals (HD; *n* = 106) and an independent ITP cohort (*n* = 13).^11^ ITP patients were grouped according to sampling time points at BL (*n* = 12 for DEX, *n* = 8 for ETB + DEX), W3 (*n* = 10 for DEX, *n* = 9 for ETB + DEX), W9 (*n* = 11 for DEX, *n* = 10 for ETB + DEX), W27 (*n* = 9 for DEX, *n* = 7 for ETB + DEX) and W53 (*n* = 6 for DEX, *n* = 6 for ETB + DEX). Statistical analysis: Pillai–Bartlett test of multivariate analysis of variance (MANOVA) of all principal components. (C) Median frequency of TRBV gene usage for the selected V gene families. (D) PCA of TRBV and TRBV‐J gene usage in the XPAG‐ITP cohort depending on the treatment arm (DEX vs. ETB + DEX) as compared to healthy individuals (HD; *n* = 106) and an independent ITP cohort (*n* = 13).^11^ (E) Median frequency of TRBV gene usage for the selected V gene families. BL, baseline; DEX, dexamethasone; ETB, eltrombopag; HD, healthy donor; ITP immune thrombocytopenia; PCA principal component analysis; TRBV T‐cell receptor beta‐chain variable genes; W3, week 3; W9, week 9; W27, week 27; W53, week 53.

Comparing the XPAG‐ITP TCR repertoire data to an independent ITP cohort^11^ revealed overlapping skewing of the V and VJ architectures (Figure 2D) and identical TRBV gene frequencies (Figure 2E).

In contrast, the BCR architecture displayed no pronounced ITP‐specific patterns. There was no inter‐patient and only marginal intra‐patient clonal overlap (Figure S1A) and no clear skewing of the V or V‐J gene architecture (Figure S1B). This was also reflected in unaltered frequencies of immunoglobulin heavy chain variable (IGHV) gene usage (Figure S1C). We also performed phylogenetic analyses in both treatment groups but could not detect BCR imprints of antigen selection that could substantiate the hypothesis of T‐cell‐primed autoantibody production in the context of the observed TCR clonality and skewing patterns (Figure S1D,E).

The limitation of our analysis is clearly the small sample size, which does not allow for in‐depth statistical analysis.

Together, we present translational data from the XPAG‐ITP trial that further establish T cells as dominant drivers of ITP pathobiology. We describe a persisting TCR repertoire skewing that is rather driven by the lack of distinct TRBV rearrangements as previously described for other autoimmune cytopenias.^11^ Patients achieving sustained remission exhibited more clonal T‐cell populations at baseline, which became more diverse over the course of treatment, indicating a gradual normalisation of the T‐cell architecture potentially linked to durable disease control.

## AUTHOR CONTRIBUTIONS

MB: study design, patient recruitment, data collection, data analysis, data interpretation and manuscript writing. MB, MR, OM, FN, AM: steering committee, patient recruitment, data collection, data analysis, data interpretation, input for the manuscript. AJJ and CS: data analysis, data interpretation, visualisation and manuscript writing. PS‐B: data collection, data analysis, visualisation and correction of the manuscript. DS: provided health donor samples. GS, MH, JC, ST, KT‐G, RR, CS, BF, MW and MS: patient recruitment, data collection and critical manuscript review. SJ, TT, CQ and FR: study design, analysis and critical manuscript input. JS, CQ and TT: clinical statistical analysis. AH and JRP: critical clinical and scientific input.

## FUNDING INFORMATION

The trial including the translational research was funded by Novartis, Swiss National Science Foundation (award 10.001.762 [to MB]) and the Mertelsmann Foundation (project grant to MB). AJJ was supported by the Jacques und Gloria Gossweiler Stiftung.

## CONFLICT OF INTEREST STATEMENT

MB received institutional research grants from Merck, BMS, Hexal, German Cancer Aid (Krebshilfe), German Research Foundation and the Federal Ministry of Education and Research as well as honoraria for lectures and advisory board meetings by Celgene, Janssen, Gilead, Merck, Roche, Amgen, Sanofi‐Aventis and BMS. She received funding for the translational research programme of the XPAG‐ITP trial from Novartis. SJ, CQ, FR and TT are Novartis Pharma GmbH employees. FR is a stock owner of Novartis Pharma GmbH. MH has received speaker honoraria and consulting fees from Novartis, Roche, Amgen, Takeda, GSK, Jazz Pharmaceuticals, Bayer Vital, Celgene, Gilead, Sanofi and Sobi. MH's travelling costs were covered by Abbvie. OM has received speaker honoraria and consulting fees from Amgen, Argenx, Grilfols, Novartis and SOBI. AM received consulting fees and payment for expert testimony from Novartis, Grifols, Sanofi, Swedish Orphan Biotech and Amgen. Furthermore, AM was supported financially to attend meetings by Grifols and Swedish Orphan Biotech, AM holds stock of Johnson & Johnson and Roche. KT‐G has received speaker honoraria and consulting fees from Amgen, Grifols, GSK, Novartis, Roche, Sanofi, Sobi and Takeda. TS received research support by Novartis, SOBI, Amgen, Argenx, Grifols, Sanofi, further consulting fees from Novartis, speaker honoraria by Novartis, Amgen, Alexion, Jannsen, AOP, SOBI, Argenx, Grifols and Astra Zeneca; TS is member of the scientific advisory board for Novartis, Amgen and Argenx; additionally, he receives funding from Celgene/BMS. All other authors declare no potential conflict of interests.

## Supporting information


Figure S1.

Data S1.

## Data Availability

Clinical data were collected in the respective clinical centres. Derived data supporting the findings of this study are available from the corresponding author MB on request.
