# Supplementary figures and images for: High pretreatment peripheral blood T‐cell receptor clonality as a predictor of prolonged response in immune thrombocytopenia
Source: Br J Haematol. 2026 Jan 4;208(2):784–8. doi: 10.1111/bjh.70310 (PMC12916192; doi:10.1111/bjh.70310)

A

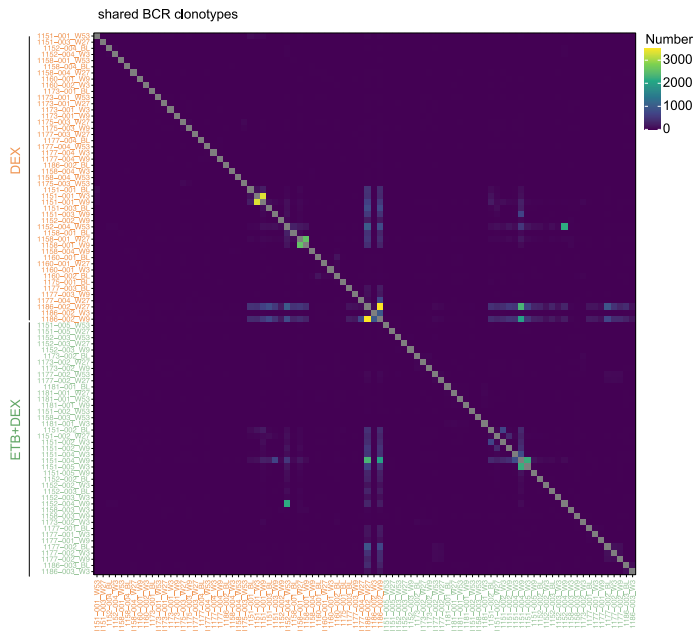

B

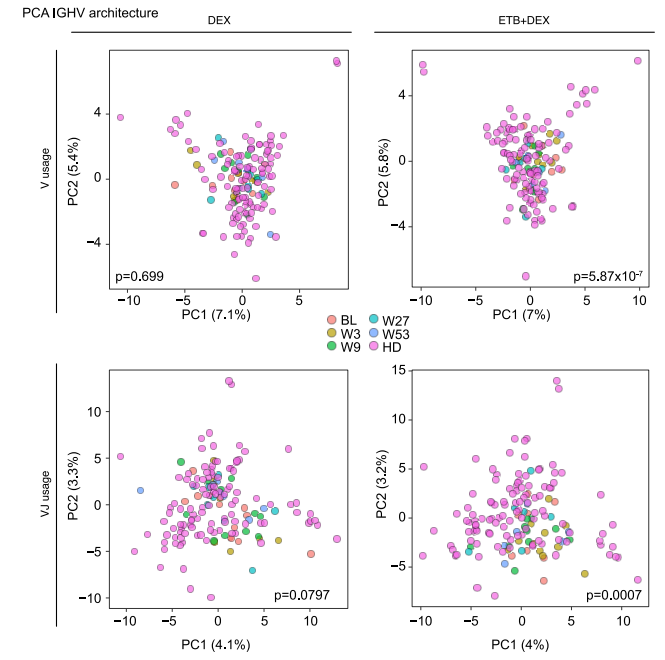

C

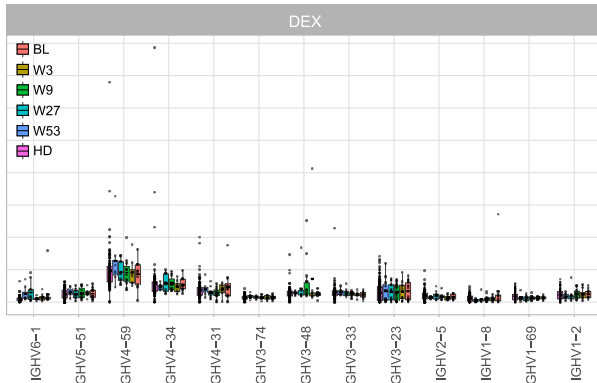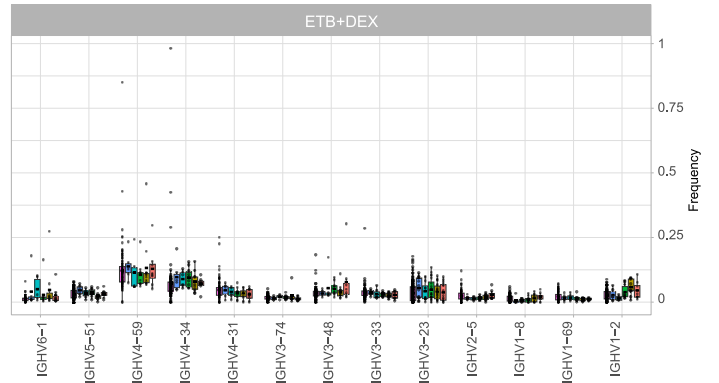

D

Top10 clonotypes DEX

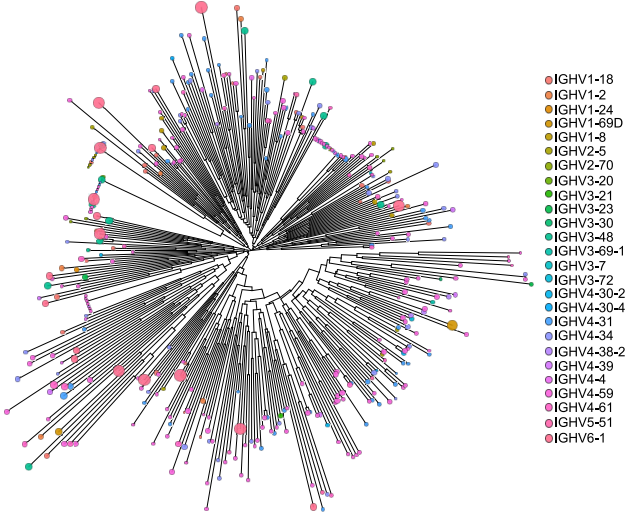

E

Top10 clonotypes ETB+DEX

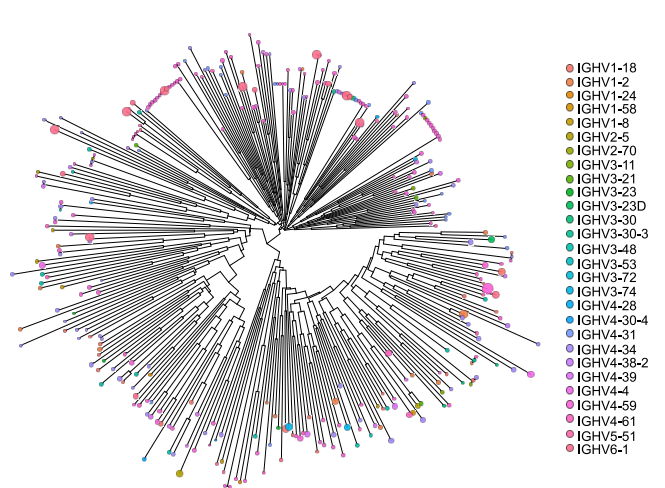

Supplement: Supplementary file 1 — Figure S1. [file BJH-208-784-s002.pdf]
