# Supplementary material for: High pretreatment peripheral blood T‐cell receptor clonality as a predictor of prolonged response in immune thrombocytopenia
Source: Br J Haematol. 2026 Jan 4;208(2):784–8. doi: 10.1111/bjh.70310 (PMC12916192; doi:10.1111/bjh.70310)
Supplement: Supplementary file 2 — Data S1. [file BJH-208-784-s001.docx]

**Supplemental material: High Pre-treatment Peripheral Blood TCR Clonality as Predictor of prolonged Response in Immune Thrombocytopenia**

**Content:**

- **Extended material and methods**
- **Figure S1**

**Material and methods**

*Clinical trial*

The XPAG-ITP (NCT04346654) was a Phase II, prospective randomized (1:1), open-label trial conducted at 25 study centres in Germany to evaluate eltrombopag (ETB) combined with dexamethasone (ETB+DEX) versus dexamethasone monotherapy (DEX) in newly diagnosed ITP, aiming to assess response durability, immune modulation, and relapse prevention. Inclusion criteria, patient demographics, randomization and outcomes are reported in Jauch *et al.* BJH 2025.

*Translational research - Biomaterial*

Patients donated 20 mL peripheral blood (collected in STRECK cell-free DNA BCT tubes) on Day 1 of week 1, 3, 9, 27 and 53 / End of study (EOS) for translational research. Blood samples from healthy donors were obtained after informed consent from the Blood Donor Centre, University Hospital of Basel and from previous studies^1-4^, complying with all national and international ethical regulations.

*Isolation of genomic DNA*

Whole blood was centrifuged 15 minutes at 2000 x g, plasma was removed and erythrocytes were lysed using red blood cell lysis buffer (C-C-Pro GmbH, Oberdorla, Germany). Genomic DNA of was isolated using the GenElute mammalian genomic DNA miniprep kit (Sigma-Aldrich, Taufkirchen, Germany) according to the manufacturer’s instructions.

*Next-generation T and B cell receptor repertoire sequencing*

To analyse blood immune cells over treatment, amplification of the T cell receptor beta chain (TRB) and immunoglobulin heavy chain (IGH) repertoire from circulating cells was performed using BIOMED2 primer pools as described elsewhere.^5^ Sequencing and de-multiplexing were performed on the Illumina MiSeq platform (600-cycle single-indexed, paired-end run, V3-chemistry) at an average coverage of 80,000 reads per sample. TRB and IGH rearrangements were aligned with MiXCR V3.0.12 using the default MiXCR library as reference for TRB sequences and the IMGT library v3 as reference for IGH sequences. TRB repertoires were proportionally normalized to 50,000 reads, IGH repertoires to 20,000 read counts. A clonotype was defined as unique complementarity-determining region 3 (CDR3) nucleotide sequence. Clonotypes with less than two read counts and nonproductive sequences were not included in downstream analyses. Analyses and data plotting were performed using tcR and immunarch packages in R (version 3.4.4), Python (version 3.11.5) as well as GraphPad Prism 7. The sequencing data set generated in this study has been deposited in the European Nucleotide Archive (ENA, ID: PRJEB7484).

**References**

1. Schultheiss C, Simnica D, Willscher E, Oberle A, Fanchi L, Bonzanni N, et al. Next-Generation Immunosequencing Reveals Pathological T-Cell Architecture in Autoimmune Hepatitis. Hepatology. 2021;73(4):1436-48.

2. Simnica D, Schliffke S, Schultheiss C, Bonzanni N, Fanchi LF, Akyuz N, et al. High-Throughput Immunogenetics Reveals a Lack of Physiological T Cell Clusters in Patients With Autoimmune Cytopenias. Front Immunol. 2019;10:1897.

3. Paschold L, Klee B, Gottschick C, Willscher E, Diexer S, Schultheiss C, et al. Rapid Hypermutation B Cell Trajectory Recruits Previously Primed B Cells Upon Third SARS-Cov-2 mRNA Vaccination. Front Immunol. 2022;13:876306.

4. Paschold L, Gottschick C, Langer S, Klee B, Diexer S, Aksentijevich I, et al. T cell repertoire breadth is associated with the number of acute respiratory infections in the LoewenKIDS birth cohort. Sci Rep. 2023;13(1):9516.

5. van Dongen J, Langerak A, Brüggemann M, Evans P, Hummel M, Lavender F, et al. Design and standardization of PCR primers and protocols for detection of clonal immunoglobulin and T-cell receptor gene recombinations in suspect lymphoproliferations: Report of the BIOMED-2 Concerted Action BMH4-CT98-3936. Leukemia. 2003;17:2257-317.


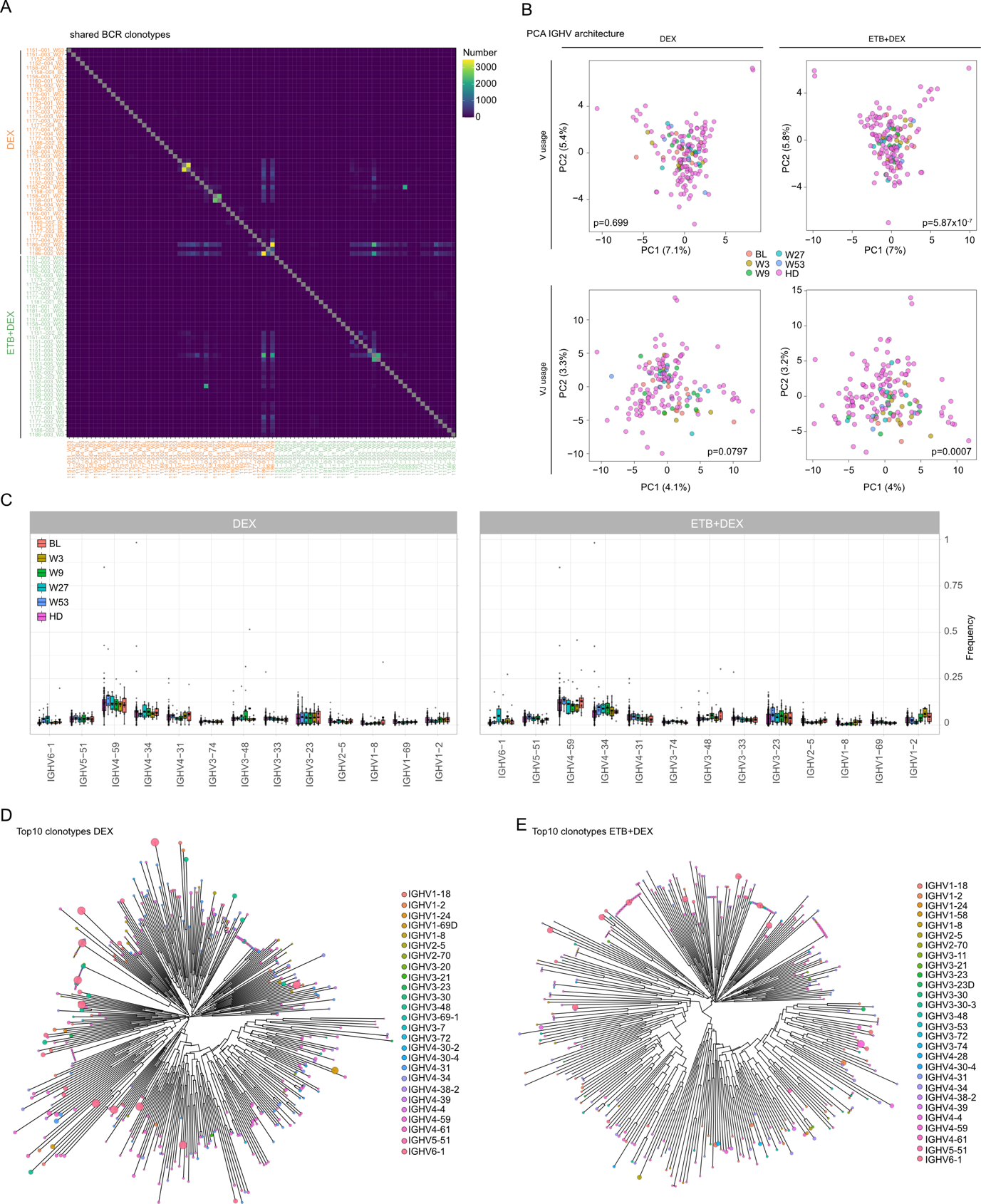


**Figure S1. B cell receptor architecture in ITP patients. (A)** Inter- and intra-patient clonal overlap of unique BCR CDR3 clonotypes displayed as a heat map. **(B)** PCA of IGHV and IGHV-J gene usage in the XPAG-ITP cohort depending on the treatment arm (DEX vs ETB+DEX) as compared to healthy individuals (HD; n=111). ITP patients were grouped according to sampling time points at BL (n=12 for DEX, n=8 for ETB+DEX), W3 (n=8 for DEX, n=11 for ETB+DEX), W9 (n=11 for DEX, n=10 for ETB+DEX), W27 (n=9 for DEX, n=6 for ETB+DEX) and W53 (n=7 for DEX, and n=6 for ETB+DEX). Statistical analysis: Pillai-Bartlett test of multivariate analysis of variance (MANOVA) of all principal components. **(C)** Median frequency of IGHV gene usage for the selected V gene families. **(D)-(E)** Phylogenetic tree for the top 10 clonotypes in all ITP patients treated with DEX **(D)** or ETB+DEX **(E)** over the course of therapy. Colours indicate IGHV family, dot sizes represent clone size. BL, baseline; DEX, dexamethasone; ETB, eltrombopag; HD, healthy donor; IGHV immunoglobulin heavy chain variable gene; W3, week 3; W9, week 9; W27, week 27; W53, week 53.
